# N-Terminal Pro-Brain Natriuretic Peptide Is a Useful Prognostic Marker in Patients with Pre-Capillary Pulmonary Hypertension and Renal Insufficiency

**DOI:** 10.1371/journal.pone.0094263

**Published:** 2014-04-21

**Authors:** Lars Harbaum, Jan K. Hennigs, Hans J. Baumann, Nicole Lüneburg, Elisabeth Griesch, Carsten Bokemeyer, Ekkehard Grünig, Hans Klose

**Affiliations:** 1 Department of Internal Medicine - Oncology, Hematology, BMT with Section Pneumology, University Medical Center Hamburg-Eppendorf, Hamburg, Germany; 2 Vera Moulton Wall Pulmonary Vascular Research Laboratories, Stanford University School of Medicine, Stanford, California, United States of America; 3 Institute of Clinical Pharmacology and Toxicology, University Medical Center Hamburg-Eppendorf, Hamburg, Germany; 4 Center for Pulmonary Hypertension, Thoraxclinic Heidelberg, University of Heidelberg, Heidelberg, Germany; VU University Medical Center, Netherlands

## Abstract

N-terminal pro-brain natriuretic peptide (NT-proBNP) is a routinely used prognostic parameter in patients with pre-capillary pulmonary hypertension (PH). As it accumulates in the presence of impaired renal function, the clinical utility of NT-proBNP in PH patients with concomitant renal insufficiency remains unclear. In a retrospective approach, patients with pre-capillary PH (group I or IV) and concomitant renal insufficiency at time of right heart catheterization (glomerular filtration rate (GFR) ≤60 ml/min/1.73 m^2^) were identified out of all prevalent pre-capillary PH patients treated at a single center. Forty patients with renal insufficiency (25.8%) were identified and matched regarding hemodynamic parameters with a control group of 56 PH patients with normal renal function (GFR >60 ml/min/1.73 m^2^). Correlations of NT-proBNP levels with hemodynamic and prognostic parameters (time to clinical worsening and overall survival) were assessed. Overall, GFR correlated inversely with NT-proBNP and had the strongest influence on NT-proBNP levels in a stepwise multiple linear regression model including hemodynamic parameters and age (r^2^ = 0.167). PH patients with renal insufficiency had significant higher levels of NT-proBNP (median: 1935 ng/l vs. 573 ng/l, p = 0.001). Nevertheless, NT-proBNP correlated with invasive hemodynamic parameters in these patients. Using higher cut-off values than in patients with preserved renal function, NT-proBNP levels were significantly associated with time to clinical worsening (>1660 ng/l, p = 0.001) and survival (>2212 ng/l, p = 0.047) in patients with renal insufficiency. Multivariate Cox’s proportional hazards analysis including established prognostic parameters, age and GFR confirmed NT-proBNP as an independent risk factor for clinical worsening in PH patients with renal insufficiency (hazard ratio 4.8, p = 0.007). Thus, in a retrospective analysis we showed that NT-proBNP levels correlated with hemodynamic parameters and outcome regardless of renal function. By using higher cut-off values, NT-proBNP seems to represent a valid clinical marker even in PH patients with renal insufficiency.

## Introduction

Pulmonary hypertension (PH) is characterized by elevated pulmonary vascular resistance (PVR) leading to right ventricular overload, hypertrophy and dilation, and eventually causing right ventricular failure and death [1]. For accurate clinical decision-making robust (surrogate) markers are warranted that reflect hemodynamic status and predict disease progression and prognosis; ideally, such markers are of non-invasive nature. In patients with pre-capillary PH, non-invasive parameters include exercise capacity, World Health Organization functional class (WHO FC), echocardiographic signs and indices including pericardial effusion, right atrium size and left ventricular eccentricity [2]–[4]. In addition, different biomarkers obtained from patients’ blood have been investigated regarding their ability to reflect disease state [5].

Brain natriuretic peptide (BNP) and the N-terminal fragments of its pro-hormone, N-terminal pro-brain natriuretic peptide (NT-proBNP), have been implicated as functional biomarkers in a variety of cardiovascular diseases [6]. Levels of BNP and NT-proBNP are elevated following ventricular impairment and reflect the severity of hemodynamic dysfunction in heart disease [6]. BNP and NT-proBNP are released from ventricular myocytes in response to mechanical stretching, e.g. due to increased chamber pressure or volume overload [7], [8]. Physiologically, BNPs are involved in volume homeostasis, regulation of blood pressure and may control structural changes of the heart muscle in a paracrine manner [7], [8]. On the contrary, NT-proBNP exhibits no or only markedly reduced biological activity [8]. The plasma concentration of NT-proBNP is influenced by various factors such as gender, age, inflammation, exercise and even diurnal variations have been reported [6], [9]. Besides an increased release, elevated NT-proBNP levels may result from altered metabolism and/or decreased elimination from the circulation. The exact mechanism of NT-proBNP elimination remains controversial [8]. However, it has been shown that impaired renal function with a decreased glomerular filtration rate (GFR) leads to accumulation of NT-proBNP and might therefore hamper its prognostic utility [10], [11].

Previous studies have shown that levels of NT-proBNP correlate with invasive hemodynamic parameters, reflect right ventricular remodeling and predict survival in patients with pre-capillary PH and normal renal function [12]–[24]. However, it remains uncertain whether NT-proBNP is a useful clinical marker in PH patients with renal dysfunction, which may occur in up to 20% of patients [16]. In fact, Leuchte et al. observed a correlation of NT-proBNP levels with right atrial pressure only in patients with PH and concomitant renal insufficiency [16].

Our analysis aimed to assess (1) the impact of renal insufficiency on NT-proBNP levels and (2) the prognostic usefulness of NT-proBNP levels in patients with pre-capillary PH (patients with pulmonary arterial hypertension, PAH, or inoperable chronic thromboembolic PH, CTEPH) and concomitant renal insufficiency. In particular, we hypothesized that NT-proBNP levels measured at time of right heart catheterization can be used to assess disease severity and predict outcome in this subgroup of patients.

## Materials and Methods

### Patients’ Selection

All outpatients with pre-capillary PH (group I or IV), who showed concomitant renal insufficiency (defined as GFR ≤60 ml/min/1.73 m^2^) at time of last right heart catheterization, were identified retrospectively out of prevalent pre-capillary PH patients treated at a tertiary PH referral centre (University Medical Centre Hamburg-Eppendorf, Hamburg, Germany). These patients were retrospectively matched in regard to hemodynamic parameters with a control group of PH patients without renal insufficiency (GFR >60 ml/min/1.73 m^2^). All patients have been diagnosed in accordance to current guidelines [25]. Right heart catheters were performed between 2003 and 2012 during routine clinical workup and after informed consent. Catheters were performed either with diagnostic or prognostic purpose. All patients with CTEPH were referred to a center for thoracic surgery specialized on pulmonary endarterectomy (Kerckhoff Clinic, Bad Nauheim, Germany). Only patients considered as inoperable were offered PAH-specific pharmacological therapy in addition to anticoagulation. Patients’ data were anonymized and de-identified prior to analyses. Anonymous data were collected in a database that was generated only for the present analysis. Analyses has been carried out in agreement with local law (Hamburgisches Krankenhausgesetz, HmbKHG, § 12) and conducted according to the principles expressed in the Declaration of Helsinki. The database is not open-source; however, we may be contacted directly to enable access to the collected anonymous data.

### Disease Parameters

For functional assessment including catheterization and laboratory findings patients were routinely admitted to our hospital for 3–5 days. All disease parameters were obtained retrospectively at the time of hospitalization for right heart catheterization (referred to as time of right heart catheterization). Hemodynamic parameters obtained from right heart catheterization included heart rate (/min, HR), mean right atrial pressure (mm Hg, RAP) mean pulmonary arterial pressure (mm Hg, mPAP) and pulmonary arterial wedge pressure (mm Hg, PAWP). Cardiac output (l/min, CO) was determined by the direct Fick method. PVR (dyne*s*cm^5^) and the cardiac index (l/min*m^2^, CI) were calculated secondary using standard formula. The 6-minute walking distance (6MWD) was obtained using a standardized protocol in accordance with the American Thoracic Society (ATS) statement (2002) [26]. Functional class according to the modified WHO classification was assessed by the attending physician. Serum-creatinine and NT-proBNP concentrations were measured using commercially available assays (Dimension Vista, Siemens Healthcare Diagnostics, IL, USA; Catalogue No. K6423A and K1033). GFR was estimated using the Modification of Diet in Renal Disease (ml/min/1.73 m^2^, MDRD) equation. Stage 3 to 5 of renal impairment (GFR ≤60 ml/min/1.73 m^2^) according to the National Kidney Foundation Practice Guidelines were defined as renal insufficiency [27]. A normalized ratio of NT-proBNP levels regarding age and gender was formed using reference data [28].

### Follow-up Assessments and Outcome Parameters

Time to clinical worsening (TTCW) was defined as the time to the first event of clinical worsening after performing right heart catheterization. Patients were evaluated at least every three months in our outpatient department. Events were retrospectively identified from patients charts and were defined as (1) decrease of more than 15% in 6MWD (2) change in PAH-specific therapy due to signs and symptoms of progressive right heart failure (either add-on or substance switch, PAH patients only), (3) lung transplantation (PAH patients only), (4) PH-related hospitalization, or (5) death due to disease. TTCW for all patients was assessed in July 2012.

All-cause mortality of patients showing renal insufficiency was obtained by reviewing the patients’ charts or by contacting the respective general physician. Overall survival was assessed in January 2014.

### Statistical Analysis

Differences of means between unpaired samples were assessed by Student t-test for parametric and Mann-Whitney-U test for non-parametric data; chi-square test was used to compare ordinal data. Spearman’s rank correlation was performed to assess correlation of non-parametric data. Time-dependent receiver-operating characteristic (ROC) analyses were performed across the ranges of NT-proBNP to assess cut-off values to predict clinical worsening and overall survival (highest sum of sensitivity and specificity). Kaplan-Meier and multivariate Cox’s proportional hazards analysis were performed to assess impact of NT-proBNP on TTCW and survival. For multivariate analysis the median of age, 6MWD and GFR in patients with renal insufficiency were used as cut-off values. All p-values were two-sided. P<0.05 was considered statistically significant. SPSS statistics 20 (IBM, Armonk, New York, United States) were used to perform all statistical analysis.

## Results

### Patients’ Characteristics

**Figure 1:**
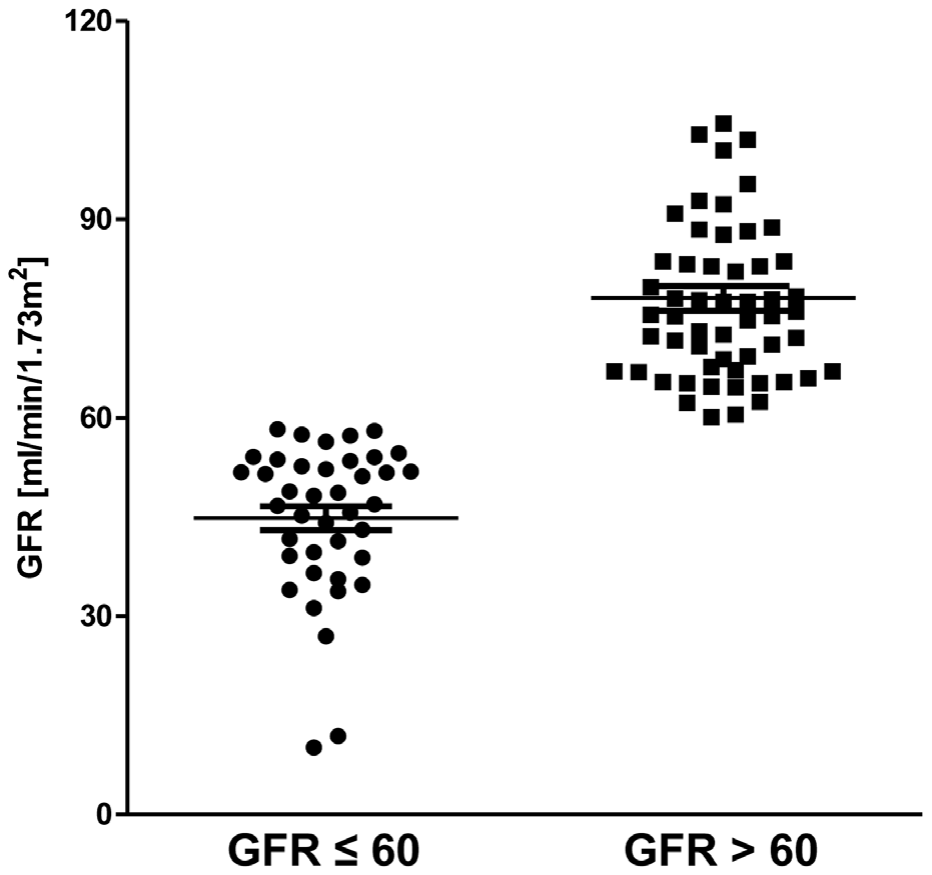
Glomerular filtration rate (GFR). Distribution of GFR (ml/min/1.73 m^2^) among patients’ groups. Mean GFR was 45±11 ml/min/1.73 m^2^ (range: 10 to 58 ml/min/1.73 m^2^) and 78±14 ml/min/1.73 m^2^ (ranging from 61 to 136 ml/min/1.73 m^2^).

Forty patients (25.8%) with pre-capillary PH and concomitant renal insufficiency were identified out of 155 prevalent pre-capillary PH patients. As a control group 56 pre-capillary PH patients without renal insufficiency were retrospectively matched regarding hemodynamic parameters. In total, 96 PH patients were included in the present analysis. Patients’ characteristics at time of right heart catheterization are shown in table 1. In patients with renal insufficiency mean GFR was 45±11 ml/min/1.73 m^2^ (range: 10 to 58 ml/min/1.73 m^2^) compared to a mean GFR of 78±14 ml/min/1.73 m^2^ (range: 61 to 136 ml/min/1.73 m^2^) in patients with normal renal function (Figure 1). Mean of serum-creatinine was 1.5±0.8 mg/dl and 0.9±0.2 mg/dl, respectively. Patients with renal insufficiency were older, reached shorter 6MWD and tended to have higher WHO FC at time of right heart catheterization (Table 1). None of patients with renal insufficiency received renal replacement therapy. All patients with CTEPH were evaluated for pulmonary endarterectomy. CTEPH patients included in the study were either considered technically not operable or denied surgery. PAH-specific therapy at time of right heart catheterization according to group of disease, i.e. PAH or CTEPH, are given in table S1. Patients received either monotherapy, combination therapy or no PAH-specific pharmacological therapy.

**Table 1:** Patients’ characteristics.

Parameter			Groups of patients		p-value
			GFR >60	GFR ≤60	
			n = 56	n = 40	
Age [yr]			60±11.7	69±10	<0.001^&^
Gender	Female		39	31	0.39^§^
	Male		17	9	
BMI [kg/m^2^]			27.1±5.8	25±5.4	0.34
PH class	IPAH		25	20	0.45^§^
	APAH		15	13	
		CTD	7	8	
		Other^#^	8	5	
	CTEPH		16	7	
WHO FC	I		3	–	0.05^§^
	II		11	3	
	III		37	36	
	IV		5	1	
6MWD [m]			366±121.8	285.6±122.8	0.002
Mean PAP [mm Hg]			42.2±14.4	40.9±13.9	0.67
RAP [mm Hg]			7.5±4.3	9.2±5.4	0.16
PAWP [mm Hg]			12±5	13±6.5	0.15
PVR [dyne*s*cm^5^]			707.2±462.7	711.2±472	0.97
CI [L/min*m^2^]			2.3±0.7	2.3±0.7	0.92

Data are presented as mean ± SD or numbers.

Comparison of means between GFR-groups are performed by Student’s T, Mann-Whitney-U^&^ or Chi-Square test^§^. CTD = connective tissue disease. ^#^ Other includes HIV, porto-pulmonary hypertension and congenital heart diseases.

### Correlation of NT-proBNP Levels with Renal Function

Overall, the level of NT-proBNP correlated inversely with GFR (r = −0.36, p<0.001). In a stepwise multiple linear regression model including mPAP, RAP, PVR, CI and age, GFR had the strongest influence on NT-proBNP levels (r^2^ = 0.167).

### Comparison of NT-proBNP Levels between PH Patients with and without Renal Insufficiency

**Figure 2:**
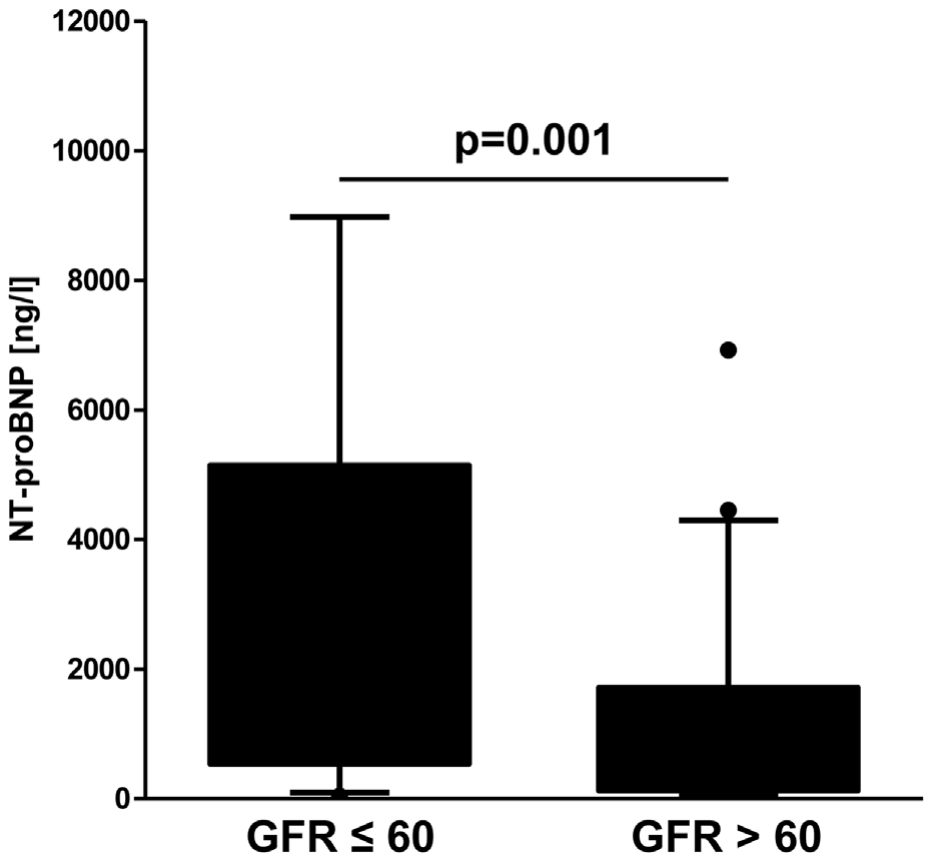
N-terminal pro-brain natriuretic peptide (NT-proBNP). In patients with renal insufficiency (defined as glomerular filtration rate (GFR) ≤60 ml/min/1.73 m^2^) the levels of NT-proBNP were significantly higher (Mann-Whitney-U-test). Median NT-proBNP concentration was 1935 ng/l (range: 44 to 14,534 ng/l) in patients with renal insufficiency and 573 ng/l (range: 47 to 6924 ng/l) in patients with normal renal function.

Levels of NT-proBNP were significantly higher in patients with renal insufficiency compared to hemodynamically matched control patients with normal renal function (p = 0.001, Mann-Whitney-U; Figure 2). Median NT-proBNP concentration was 1935 ng/l (range: 44 to 14,534 ng/l) in patients with renal insufficiency and 573 ng/l (range: 47 to 6924 ng/l) in patients with normal renal function. Moreover, NT-proBNP levels were significantly increased in patients with renal insufficiency when comparing only patients with WHO FC III/IV (p = 0.017, Mann-Whitney-U) and of older age (older than the mean age of 64 years, p<0.001, Mann-Whitney-U). However, older patients with renal insufficiency had more impaired hemodynamics compared to older patients with preserved renal function (mPAP 38±13 vs. 34±8 mmHg, p = 0.003; PVR 714±523 vs. 503±203 dyne*s*cm^5^, p = 0.004; T-tests).

### Correlation of NT-proBNP with Hemodynamic Parameters

In patients with normal renal function, NT-proBNP levels correlated with all hemodynamic parameters but RAP. In patients with renal insufficiency, NT-proBNP levels were significantly associated with all hemodynamic parameters including RAP, PVR and CI. Although statistical significant, correlations were generally moderate. An age and gender normalized ratio of NT-proBNP could not markedly improve correlation in patients with or without renal insufficiency (Table S2).

### Prediction of Clinical Worsening by NT-proBNP Levels

During a mean follow-up time of 20.6 months (range: 0 to 106 months) clinical worsening occurred in 44 out of 96 (46%) patients at a mean time of 8.8 months (range: 0 to 84 months). Causes for clinical worsening were as following: Decrease of more than 15% in 6MWD (n = 16 cases, 36%), hospitalization for progressive right heart failure (n = 14 patients, 32%), escalation of PAH-specific medication (n = 14, 32%) and two patients died during follow-up assessment regarding TTCW. From this cohort, no patient underwent lung transplantation during the follow-up period.

**Figure 3:**
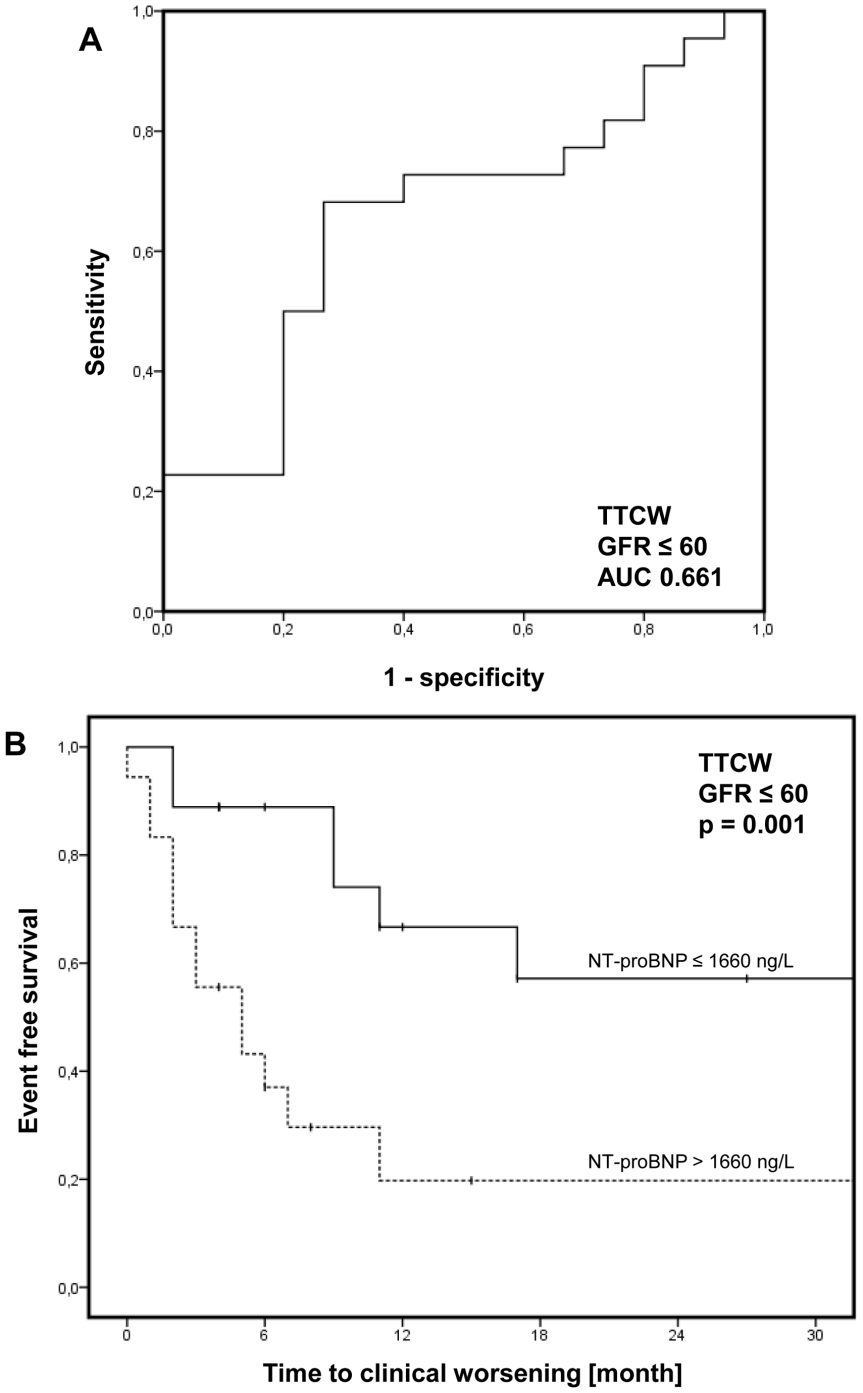
Time to clinical worsening (TTCW). Receiver operating characteristic (ROC) analysis to determine the cut-off value in patients with renal insufficiency (defined as glomerular filtration rate (GFR) ≤60 ml/min/1.73 m^2^; **A**). In Kaplan-Meier analysis, higher levels of n-terminal pro-brain natriuretic peptide (NT-proBNP) were significantly associated with early clinical worsening (**B;** p = 0.001, log-rank).

Cut-off NT-proBNP values to predict TTCW were assessed by ROC analysis. Using a cut-off value of 1292 ng/l in patients with normal renal function (sensitivity 50%, specificity 83%; AUC = 0.603; Figure S1) higher levels of NT-proBNP were significantly associated with early clinical worsening in Kaplan-Meier analysis (p = 0.02, log-rank; Figure S1). In PH patients with renal insufficiency a cut-off value of 1660 ng/l (sensitivity 68%, specificity 73%; AUC = 0.661; Figure 3A) was identified and NT-proBNP levels above this threshold were significantly associated with unfavorable clinical outcome (p = 0.001, log-rank; Figure 3B). Of note, no difference occurred regarding GFR if patients were stratified according to NT-proBNP cut-offs. GFR was 48±7 in patients with renal insufficiency and NT-proBNP levels beneath or equal to and 43±12 above 1660 ng/l (p = 0.11, t-test).

Most interestingly, among patients with renal insufficiency, NT-proBNP levels above 1660 ng/l were independently associated with early clinical worsening in a multivariate Cox’s proportional hazards analysis including age, gender, renal function (GFR) as wells as non-invasive prognostic parameter such as 6MWD and WHO FC (Hazard ratio 4.8, 95% CI 1.6–14.9, p = 0.007; Table 2).

**Table 2:** Multivariate Cox’s proportional hazards analysis assessing the predictive value of n-terminal pro-brain natriuretic peptide (NT-proBNP) levels on clinical worsening in 40 PH patients with concomitant renal insufficiency (defined as glomerular filtration rate (GFR) ≤60 ml/min/1.73 m^2^) in a model with further established non-invasive parameters, age and renal function.

Parameter	Hazard ratio	95% confidence interval		p-value
		Lower	Upper	
Age >71 yr	1.3	0.5	3.8	0.58
Male gender	0.7	0.1	3.5	0.66
GFR <48 ml/min/1.73 m^2^	1.5	0.5	4.5	0.44
6MWD <279 m	2.3	0.7	7,4	0.17
WHO FC	15.1	1.2	196.1	0.038
NT-proBNP>1660 ng/l	4.8	1.6	14.9	0.007

Median of age, 6MWD and GFR in patients with renal insufficiency were used as cut-off values.

### Prediction of Survival by NT-proBNP levels in PH Patients with Renal Insufficiency

**Figure 4:**
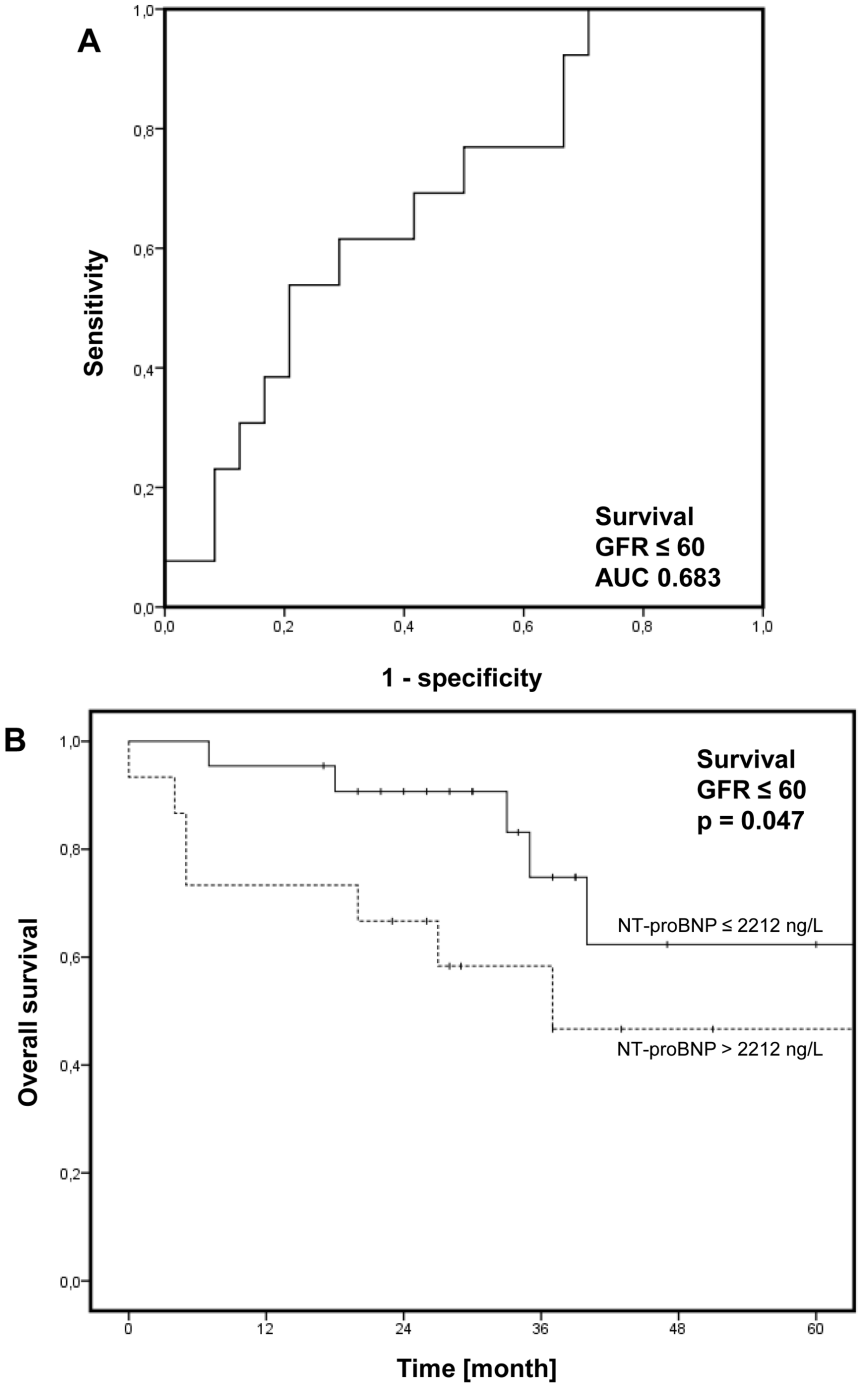
Overall Survival. Receiver operating characteristic (ROC) analysis to determine the cut-off value in patients with renal insufficiency (defined as glomerular filtration rate (GFR) ≤60 ml/min/1.73 m^2^; **A**). In Kaplan-Meier analysis, higher levels of n-terminal pro-brain natriuretic peptide (NT-proBNP) were significantly associated with poor survival (**B;** p = 0.047, log-rank).

During a mean follow-up time of 39 months (range: 0 to 126) 13 out of 40 (33%) patients with renal insufficiency died. In ROC analysis, a NT-proBNP cut-off value of 2212 ng/l (sensitivity 62%, specificity 71%; AUC = 0.68; Figure 4A) was identified and levels above this threshold were significantly associated with unfavorable overall survival (p = 0.047, log-rank; Figure 4B). One-year survival rates of patients were 95% and 73% and five-year survival rates 67% and 43%. Again, GFR did not differ between patients with higher (>2212 ng/l; 43±7 ml/min/1.73 m^2^) and lower NT-proBNP levels (≤2212 ng/l; 47±13 ml/min/1.73 m^2^, p = 0.21, t-test).

In a multivariate Cox’s proportional hazards analysis NT-proBNP levels above 2212 ng/l were not independently associated with survival if age, gender, renal function (GFR), 6MWD as well as WHO FC were included in the analysis (Hazard ratio 2.226, 95% CI 0.61–8.016, p = 0.22).

## Discussion

According to our data in pre-capillary PH patients, NT-proBNP levels are significantly affected by the renal function and accumulate in the presence of renal impairment. Nevertheless, in pre-capillary PH patients with concomitant renal insufficiency NT-proBNP levels at time of right heart catheterization correlate with relevant invasive hemodynamic parameters and are associated with clinical worsening as well as survival.

Renal insufficiency displays a crucial co-morbidity in patients with pre-capillary PH and may develop due to hemodynamic failure with low cardiac output or may exist independently [16]. In the US-based observational REVEAL registry (Registry to EValuate Early And Long-term PAH), for instance, renal insufficiency was found in only 4% of patients at time of enrolment. However, it was significantly associated with unfavorable one-year overall mortality (hazard ratio 1.9) [29]. Others have observed renal insufficiency more frequently in around 20% of PH patients [16]. Furthermore, two studies have reported that a preserved renal function was associated with better in-hospital and short-term survival in PH patients who have been hospitalized due to right heart failure (HF) [30], [31].

The elimination of NT-proBNP from the circulation lacks known active clearance mechanisms and relies presumably on passive excretion by organ beds with large degrees of blood flow including the kidneys [8]. In patients with acute or chronic left HF it seems evident that NT-proBNP levels are higher in those with renal dysfunction suggesting that the clearance depends on renal elimination [32]. Despite this association, it has been shown that NT-proBNP levels stratify outcome in patients with chronic left HF and concomittant renal dysfunction [33]. In addition, Hori et al. have reported an even better survival stratification by NT-proBNP as compared to BNP in chronic left HF patients with severe renal insufficiency (GFR <30 ml/min/1.73 m^2^) [34]. In the latter study cut-off values varied widely based on renal function (range: 258 to 5809 ng/l) [32], [34]. Thus, on an individual level uncertainty remains in the interpretation of NT-proBNP in patients with renal dysfunction.

In patients with pre-capillary PH, NT-proBNP levels have been demonstrated to correlate with invasive hemodynamic parameters such as RAP, mPAP, PVR and CI [12]–[14], [16], [17], [19], [20], [24], [35], as well as non-invasive parameters of disease severity such as 6MWD and WHO FC [14], [17], [19]–[21], [24]. Moreover, base-line NT-proBNP values have been shown to stratify patients’ survival in numerous studies [14]–[16], [20], [23]. Mathai et al. have reported an association between NT-proBNP levels and survival in a cohort of 98 PAH patients independent of renal function [19]. Estimated renal function, however, was mostly normal in these patients (mean GFR of 82±31 ml/min/1.73 m^2^) [19]. The cut-off values for NT-proBNP in studies assessing the prognostic utility of NT-proBNP in PH patients have ranged between 1256 to 1800 ng/l. Although larger outcome studies are still required to verify the optimal cut-off level, a NT-proBNP level below 1400 ng/l seems useful for identification of PAH patients with favorable prognosis [25]. Furthermore, a “normalization” of BNP or NT-proBNP level has recently been suggested as a potential treatment goal in PAH [36].

We show that renal insufficiency can be observed in a significant number of patients with pre-capillary PH. In these patients elevation of NT-proBNP due to renal dysfunction has to be taken into account if NT-proBNP level is used for risk-stratification or even as treatment goal. According to our data, differences in NT-proBNP as predictor in ROC analysis regarding short-term clinical worsening are relatively small between patient with (1292 ng/l) and without renal insufficiency (1660 ng/l). In contrast, in patients with renal insufficiency a higher cut-off (2212 ng/l) is required for stratification of survival.

There are some limitations to our study - first and foremost the study’s retrospective design: Observational periods and medication history (i.e. use, type, and duration) are varying. Furthermore, patients with CTEPH and PAH may behave differently if PAH-specific medication is given. However, patients with inoperable CTEPH have been suggested to show overlapping pathophysiological features with PAH patients, e.g. small vessel arteriopathy regardless of thromboembolism [37]. The number of included patients in our study is relatively small. Thus, our multivariate analyses may perform in a different way than others (e.g. REVEAL). The measurement of renal function at time of right heart catheterization displays a “snap-shot” and variations over time are possible and very likely. Nevertheless, our observation reflects routine clinical management of patients with these rare diseases in a PH referral centre.

In a retrospective study with two hemodynamically matched groups of PH patients with and without renal insufficiency, we show that, although NT-proBNP levels are significantly affected by renal function, this marker correlates with relevant hemodynamic parameters and is associated with clinical outcome and survival even in patients with renal insufficiency if higher cut-off are used. Thus, in these patients NT-proBNP seems to represent valid clinical marker and facilitate clinical decision-making.

## Supporting Information

Figure S1 Time to clinical worsening (TTCW): Receiver operating characteristic (ROC) analysis to determine the cut-off value in patients with preserved renal function (defined as glomerular filtration rate (GFR) >60 ml/min/1.73 m2; **A**). In Kaplan-Meier analysis, higher levels of n-terminal pro-brain natriuretic peptide (NT-proBNP) were significantly associated with early clinical worsening (**B**).
(PDF)

Table S1 Patients’ PAH-specific medication at time of right heart catheterization.
(PDF)

Table S2 Correlation of n-terminal pro-brain natriuretic peptide (NT-proBNP) with hemodynamic parameters in PH patients with and without renal insufficiency.
(PDF)
