# Supplementary material for: N-Terminal Pro-Brain Natriuretic Peptide Is a Useful Prognostic Marker in Patients with Pre-Capillary Pulmonary Hypertension and Renal Insufficiency
Source: PLoS One. 2014 Apr 21;9(4):e94263. doi: 10.1371/journal.pone.0094263 (PMC3994009; doi:10.1371/journal.pone.0094263)
Supplement: Figure S1 — Time to clinical worsening (TTCW): Receiver operating characteristic (ROC) analysis to determine the cut-off value in patients with preserved renal function (defined as glomerular filtration rate (GFR) >60 ml/min/1.73 m2; A). In Kaplan-Meier analysis, higher levels of n-terminal pro-brain natriuretic peptide (NT-proBNP) were significantly associated with early clinical worsening (B). (PDF) [file pone.0094263.s001.pdf]

Figure S1

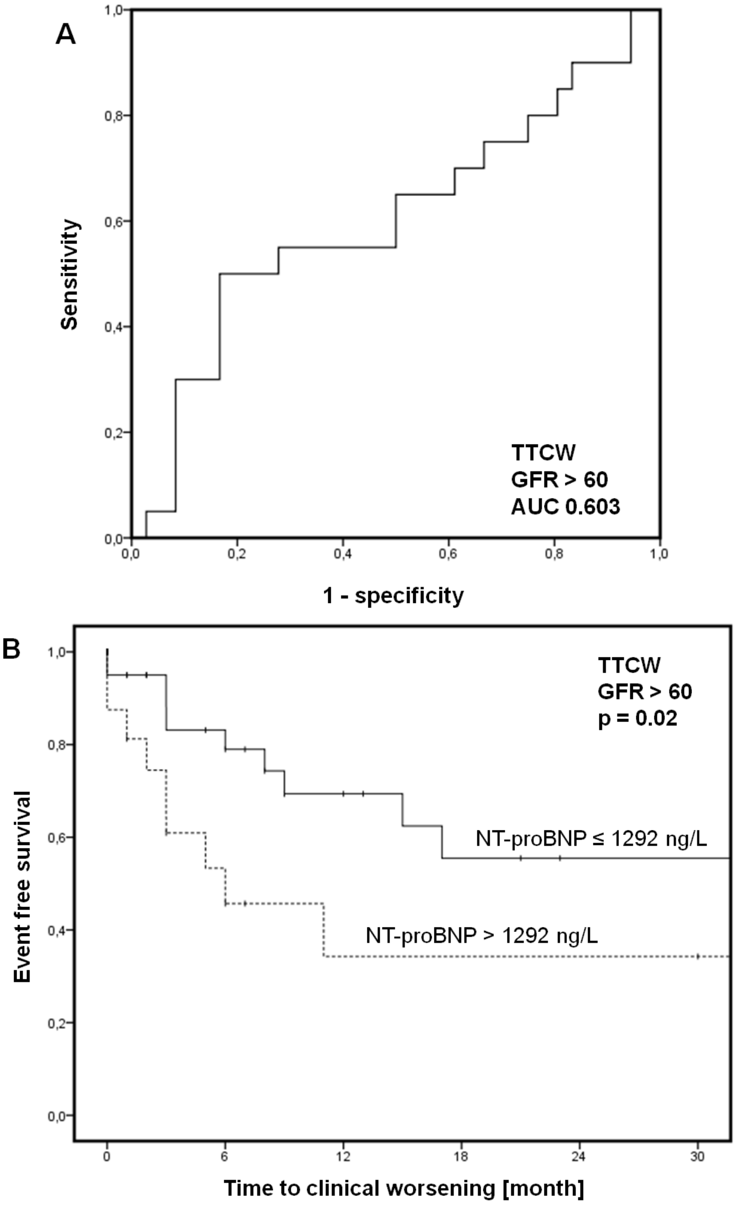

Figure S1 **Time to clinical worsening (TTCW):** Receiver operating characteristic (ROC) analysis to determine the cut-off value in patients with preserved renal function (defined as glomerular filtration rate (GFR) > 60 ml/min/1.73 m^2; **A**). In Kaplan-Meier analysis, higher levels of n-terminal pro-brain natriuretic peptide (NT-proBNP) were significantly associated with early clinical worsening (**B**).
