# Supplementary material for: N-Terminal Pro-Brain Natriuretic Peptide Is a Useful Prognostic Marker in Patients with Pre-Capillary Pulmonary Hypertension and Renal Insufficiency
Source: PLoS One. 2014 Apr 21;9(4):e94263. doi: 10.1371/journal.pone.0094263 (PMC3994009; doi:10.1371/journal.pone.0094263)
Supplement: Table S1 — Patients’ PAH-specific medication at time of right heart catheterization. (PDF) [file pone.0094263.s002.pdf]

Table S1 Patients' PAH-specific medication at time of right heart catheterization.

Medication	All patients	PAH	CTEPH
**Patients with GFR >60 [ml/min/1.73 m^2]**			
Oral endothelin receptor antagonists	33/56 (59%)	25/38 (66%)	8/17 (47%)
Oral phosphodiesterase type 5 inhibitors	24/56 (43%)	22/38 (58%)	6/17 (35%)
Inhaled prostacyclin	9/56 (16%)	9/38 (24%)	-
**Patients with GFR ≤60 [ml/min/1.73 m^2]**			
Oral endothelin receptor antagonists	30/40 (75%)	22/31 (71%)	2/8 (25%)
Oral phosphodiesterase type 5 inhibitors	33/40 (83%)	30/31 (65%)	3/8 (38%)
Inhaled prostacyclin	8/40 (20%)	8/31 (26%)	-
