# Supplementary material for: N-Terminal Pro-Brain Natriuretic Peptide Is a Useful Prognostic Marker in Patients with Pre-Capillary Pulmonary Hypertension and Renal Insufficiency
Source: PLoS One. 2014 Apr 21;9(4):e94263. doi: 10.1371/journal.pone.0094263 (PMC3994009; doi:10.1371/journal.pone.0094263)
Supplement: Table S2 — Correlation of n-terminal pro-brain natriuretic peptide (NT-proBNP) with hemodynamic parameters in PH patients with and without renal insufficiency. (PDF) [file pone.0094263.s003.pdf]

Table S2 Correlation of n-terminal pro-brain natriuretic peptide (NT-proBNP) with hemodynamic parameters in PH patients with and without renal insufficiency.

Parameters	NT-proBNP [ng/L]	Normalized NT-proBNP ratio
**Patients with GFR >60 [ml/min/1.73 m^2] (n=56)**		
Mean PAP [mm Hg]	0.454; <0.001	0.404; 0.002
RAP [mm Hg]	0.244; 0.2	0.245; 0.2
PVR [dyne*s*cm^5]	0.513; <0.001	0.474; <0.001
CI [L/min*m^2]	-0.370; 0.006	-0.386; 0.004
**Patients with GFR ≤60 [ml/min/1.73 m^2] (n=40)**		
Mean PAP [mm Hg]	-0.057; 0.74	0.006; 0.97
RAP [mm Hg]	0.410; 0.016	0.385: 0.018
PVR [dyne*s*cm^5]	0.343; 0.044	0.318; 0.052
CI [L/min*m^2]	-0.457; 0.009	-0.452; 0.007

Data are presented as R-value; p-value of Spearmen’s rank correlation.
